# Parenting Profiles in Chinese Preschool Families: Parental Emotional Intelligence and Children’s Social Competence

**DOI:** 10.3390/bs16081395

**Published:** 2026-08-14

**Authors:** Songliang Li, Xiaosong Gai

**Affiliations:** 1 School of Psychology, Northeast Normal University, 5268 Renmin Street, Changchun 130024, China; songlian@asu.edu; 2 Research Center of Mental Health Education in Northeast Normal University, Key Research Institute of Humanities and Social Science in Universities in Jilin Province, Northeast Normal University, Changchun 130024, China

**Keywords:** parental emotional intelligence, parenting practices, parenting profiles, social competence, preschool children, latent profile analysis, emotion socialization

## Abstract

Parental emotional intelligence (EI) has been associated with children’s socio-emotional development, yet less is known about how parental EI is associated with naturally occurring configurations of parenting practices. This cross-sectional study investigated two complementary statistical indirect associations connecting parental EI to preschoolers’ social competence using a sample of 13,398 Chinese families: a variable-centered pathway via positive and negative parenting practices, and a person-centered pathway via distinct parenting profiles. Exploratory latent profile analysis yielded four sample-specific parenting configurations: high-positive/low-negative (27.49%), near-average (55.19%), low-positive/elevated-negative (9.16%), and mixed slightly positive/high-negative (8.16%). Parental EI exhibited a positive concurrent association with children’s overall social competence. Positive and negative parenting statistically accounted for part of this bivariate association at the variable level, while parenting profile membership provided complementary person-centered information about differences in child social competence. Family climate was incorporated as an auxiliary contextual correlate and was not framed as a core predictor, mediator, or moderator within the focal model. Given the cross-sectional single-wave design and caregiver-reported data for all key constructs, all observed patterns should only be interpreted as correlational associations, rather than empirical support for causal mediation.

## 1. Introduction

Preschool children’s social competence is a foundational developmental capacity that enables children to participate effectively in peer interaction, classroom routines, and family life. During early childhood, children are expected not only to cooperate, share, help others, and follow group expectations, but also to regulate emotional responses and understand themselves in relation to others. These capacities are closely associated with later peer acceptance, school readiness, behavioral adjustment, and socio-emotional well-being (Denham et al., 2003; Halberstadt et al., 2001; McDowell et al., 2002). Importantly, social competence does not develop as an isolated individual trait. Rather, it emerges through repeated interactions in which children observe how emotions are expressed, how conflicts are resolved, and how interpersonal expectations are negotiated within close relationships (Lunkenheimer et al., 2020; Morris et al., 2007, 2017).

The family is one of the earliest and most influential contexts in which children acquire these emotional and social capacities. Emotion socialization theory proposes that parents shape children’s emotional and social adjustment through their emotional expressiveness, responses to children’s emotions, emotion-related conversations, and the broader emotional tone of family interaction (Eisenberg et al., 1998; Morris et al., 2007). Supportive emotion socialization may help children understand emotional cues, regulate affective arousal, and use socially appropriate strategies in peer and family contexts. In contrast, harsh, dismissive, or inconsistent emotional responses may limit children’s opportunities to practice adaptive regulation and constructive social behavior. Recent intervention and review evidence further suggests that parent emotion socialization practices are relevant to young children’s emotional competence and behavioral adjustment (England-Mason et al., 2023; Havighurst et al., 2010, 2022). These findings indicate that parents’ emotional functioning may be meaningfully associated with children’s social development, particularly during the preschool years when self-regulatory capacities remain strongly scaffolded by adults.

### 1.1. Parental Emotional Intelligence and Children’s Social Competence

Parental emotional intelligence (EI) provides one useful framework for understanding parents’ emotional functioning in family contexts. EI is generally defined as a set of abilities or self-perceived competencies involved in perceiving, understanding, using, and regulating emotions (Mayer et al., 2016; Salovey & Mayer, 1990). In daily caregiving, parents with higher EI may be better able to notice children’s emotional signals, manage their own frustration during difficult interactions, and respond to children in ways that support emotional understanding and social learning. A systematic review found that parental EI is generally associated with more favorable child mental health and adjustment outcomes, although the literature remains limited by cross-sectional designs and heterogeneous measurement approaches (Romero González et al., 2021). Although EI was initially studied mainly as an individual difference related to personal and interpersonal adaptation, evidence increasingly suggests that parents’ EI may have intergenerational significance because it shapes the emotional models and regulatory resources available to children (Brackett et al., 2006). A study reported that parenting style mediated associations between parents’ EI and adolescent aggression, indicating that parental EI may influence child outcomes through parenting behavior (Batool & Bond, 2015). Other research has shown that parental communication and EI are related to child-to-parent violence and broader family functioning, again implying that emotional competence is expressed through interactional patterns rather than operating only as an internal trait (López-Martínez et al., 2019).

More recent research has also reported associations between parents’ and offspring’s emotional abilities, suggesting that emotional competence may be reflected across generations through family interaction processes (Cabello et al., 2023; Sánchez-Núñez et al., 2020). Studies on family EI and parent–child emotional functioning suggest that emotionally competent parents are more likely to support open communication and adaptive emotional expression, both of which are relevant to children’s mental health and social adaptation (Șițoiu & Pânișoară, 2023). Hence, parental emotional intelligence may be associated with children’s outcomes.

Nevertheless, parental EI is unlikely to be the immediate developmental context that children experience directly. Children do not encounter parental EI as an abstract psychological resource; rather, they encounter it through parents’ daily behaviors, emotional reactions, and patterns of guidance. The process model of parenting suggests that parenting behavior is shaped by parental psychological resources, child characteristics, and contextual stressors (Belsky, 1984). From this perspective, parental EI may be associated with children’s social competence because emotionally competent parents are more likely to show warmth, autonomy support, and structure, and less likely to respond with rejection, coercion, or chaotic discipline. This interpretation is consistent with evidence that positive parenting is related to children’s effortful control, empathy, prosocial behavior, and lower externalizing problems, whereas harsh or inconsistent parenting is related to poorer socio-emotional functioning (Eisenberg et al., 2005; Morris et al., 2017; Taylor et al., 2013).

### 1.2. Parenting Practices as Behavioral Expressions of Parental Emotional Intelligence

Parenting practices represent the behavioral channels through which family emotional resources are translated into children’s daily developmental experiences. Classical parenting theory conceptualizes parenting style as the emotional and regulatory context in which specific parenting behaviors acquire meaning (Darling & Steinberg, 1993). Emotion-related parenting studies further show that supportive reactions and parent–child conversations about emotion are linked to children’s emotion management, prosocial behavior, and lower aggression (Garner et al., 2008; Miller-Slough et al., 2016). By contrast, harsh, rejecting, or inconsistent parenting can interfere with children’s regulation and peer competence, partly because children may internalize coercive interaction patterns or become less able to manage frustration in social settings (McDowell et al., 2002; Chan et al., 2009). These findings suggest that children’s social competence should be examined not only in relation to global parental traits, such as EI, but also in relation to the concrete parenting patterns that structure children’s everyday social learning.

Most existing studies have examined parenting through variable-centered models, estimating the separate contribution of specific dimensions such as warmth, structure, rejection, or coercion. This approach is valuable because it clarifies which parenting practices are associated with children’s outcomes. However, it may not fully capture the way parenting is organized in real families. Children experience parenting as a pattern of behaviors rather than as isolated dimensions. A parent may be both warm and highly controlling, structured but emotionally rejecting, or low in both support and harsh control. These combinations are theoretically important because the meaning of one parenting behavior may depend on the broader configuration in which it appears. For example, high structure may be experienced differently when it is embedded in warmth than when it is accompanied by coercion or rejection.

This limitation has led to increasing interest in person-centered approaches to parenting and emotion socialization. Unlike variable-centered models, which ask whether one parenting dimension predicts an outcome after controlling for other dimensions, person-centered models ask how multiple parenting practices are organized within families. Recent latent profile and profile-based studies have shown that parents can be grouped into distinct emotion socialization or parenting configurations, and that these configurations are differentially associated with parental functioning and child adjustment (Arikan & Kumru, 2023; Howe & Zimmer-Gembeck, 2022; King et al., 2023; Trevethan et al., 2021). These studies suggest that parenting profiles may reveal patterns that are not visible when positive and negative parenting are examined only as separate linear predictors.

### 1.3. From Parenting Practices to Parenting Profiles: A Person-Centered Perspective

A person-centered approach may be particularly useful for studying parenting in Chinese preschool families. In many Chinese family contexts, parental involvement, structure, academic or behavioral expectations, and control may coexist in ways that are not fully captured by Western-derived positive-versus-negative parenting distinctions (Chao, 2000; Friedlmeier et al., 2011). This does not mean that high control is uniformly adaptive or maladaptive across cultural contexts. Rather, it suggests that the same family may show both supportive and controlling features, and that these mixed configurations require empirical identification rather than theoretical assumption. Latent profile analysis (LPA) is therefore well suited to the present research question because it allows warmth, autonomy support, structure, rejection, chaos, and coercion to be examined as co-occurring parenting configurations. In this sense, LPA is not merely an additional statistical procedure, but a theoretically motivated strategy for understanding how multiple parenting practices are organized in families.

Family climate is also relevant to this framework, but its analytic role requires careful clarification. A warm, cohesive, and communicative family climate may provide a broader emotional context in which parental EI and parenting practices are expressed. Prior research suggests that family emotional processes are important for children’s regulation and adjustment (Morris et al., 2007; Stocker et al., 2007). However, family climate overlaps conceptually with both parental emotional functioning and parenting behavior. If family climate is simultaneously treated as a predictor, mediator, moderator, and background condition, the theoretical model becomes difficult to interpret. Therefore, the present study treats family climate as a supplementary contextual correlate rather than as the focal independent variable or a primary mediator. This positioning allows the study to acknowledge the broader emotional family environment while keeping the central model focused on parental EI, parenting practices, parenting profiles, and children’s social competence.

### 1.4. The Present Study

Collectively, existing literature reveals three key limitations requiring further empirical investigation: (a) few studies systematically test the mediating role of holistic parenting configurations in the link between parental EI and preschool children’s social competence; (b) variable-centered linear models cannot fully capture the mixed supportive-controlling parenting patterns prevalent in Chinese families; (c) cross-cultural evidence on this mediational chain is dominated by Western samples, with limited research focused on Chinese preschool populations. To address these gaps, the current study investigates interrelations among parental emotional intelligence, family climate, person-centered parenting profiles, and multidimensional social competence among a large sample of Chinese preschool families.

The present study examined the associations among parental emotional intelligence, family climate, parenting profiles, and preschool children’s social competence in a large sample of Chinese families. Following the structure of the existing literature and the measurement framework of the current project, children’s social competence was conceptualized as a multidimensional construct including positive social behavior, emotion regulation, self-awareness, and group norm adaptation. Parenting was examined using six dimensions: warmth, autonomy support, structure, rejection, chaos, and coercion. This design allowed the study to move beyond a simple positive-versus-negative parenting distinction and to identify naturally occurring parenting profiles through a person-centered approach.

First, this model was grounded in evidence that supportive and unsupportive parenting practices are differentially associated with children’s emotion regulation, prosocial behavior, and peer competence (Denham et al., 2003; Garner & Estep, 2001; Morris et al., 2017). Second, an exploratory person-centered model tested whether parenting profile membership statistically accounted for part of the same association. This model was grounded in person-centered parenting research showing that family-level configurations of parenting may provide information beyond isolated parenting dimensions (Arikan & Kumru, 2023; King et al., 2023).

Based on emotion socialization theory and previous empirical findings, four expectations guided the analyses: (1) parental EI was expected to be positively associated with children’s overall social competence; (2) parental EI was expected to be associated with more adaptive parenting practices, and positive and negative parenting were expected to statistically account for part of the EI-social competence association; (3) because the exact number and composition of parenting profiles could not be specified a priori, LPA was treated as exploratory; nevertheless, profiles characterized by higher warmth, autonomy support, and structure and lower rejection, chaos, and coercion were expected to show higher child social competence than less adaptive profiles; (4) parenting profile membership was expected to statistically account for part of the association between parental EI and child social competence. Socioeconomic status and child demographic characteristics were included as covariates, and child gender was examined as an exploratory moderator given prior evidence of gender differences in temperament, emotional expression, and peer relationship processes (Else-Quest et al., 2006; Rose & Rudolph, 2006). Because all variables were measured concurrently, these expectations were tested as cross-sectional associations rather than causal developmental pathways.

## 2. Materials and Methods

### 2.1. Participants

This study used a cross-sectional questionnaire design to examine the associations among parental emotional intelligence, family climate, parenting profiles, and preschool children’s social competence. Participants were recruited from 48 public kindergartens located in seven provincial-level regions of mainland China, including Jilin Province, Tianjin Municipality, Henan Province, Shaanxi Province, Guizhou Province, Zhejiang Province, and Guangdong Province. Data were collected between August and October 2022 through an online survey platform (Wenjuanxing; https://www.wjx.cn/). Parents or primary caregivers were invited to complete the questionnaire according to their actual parenting experiences and their observations of the target child. Participation was voluntary, and respondents were informed that their responses would be kept confidential and used only for research purposes.

A total of 15,627 caregivers initially completed the questionnaire. The initial respondent pool included 3495 fathers, 12,026 mothers, and 106 other primary caregivers. To improve data quality, validity-check items embedded in the questionnaire were used to identify careless or invalid responses. After excluding respondents who failed the lie-detection items or reported that they had not answered the questionnaire carefully, 13,398 participants remained in the final sample, resulting in an exclusion rate of 14.3%. In the final sample, 7058 children were boys (52.7%) and 6340 were girls (47.3%). The mean age of the children was 4.42 years (SD = 1.13). Most respondents lived in urban areas (*n* = 11,092, 82.8%), whereas 2306 lived in rural areas (17.2%). Mothers were the most common respondents (*n* = 10,419, 77.8%), followed by fathers (*n* = 2899, 21.6%) and other caregivers (*n* = 80, 0.6%). Most children lived in two-parent families (*n* = 13,199, 98.5%), and 199 children lived in single-parent families (1.5%).

### 2.2. Measures

#### 2.2.1. Emotional Intelligence Scale

Parental emotional intelligence was measured using the Wong and Law Emotional Intelligence Scale (Wong & Law, 2002). The WLEIS is a 16-item self-report measure designed to assess four dimensions of emotional intelligence: self-emotion appraisal, others’ emotion appraisal, emotion regulation, and emotion use. Items were rated on a 7-point Likert scale ranging from 1 (strongly disagree) to 7 (strongly agree), with higher scores indicating higher parental emotional intelligence. The Chinese version of the scale has been widely used in prior research and is consistent with the four-factor structure proposed in the original measure. The four-factor WLEIS model showed strong comparative fit and low residual misfit but elevated RMSEA: chi-square (98) = 27,011.278, *p* < 0.001, Comparative Fit Index (CFI) = 0.961, Tucker–Lewis Index (TLI) = 0.953, Root Mean Square Error of Approximation (RMSEA) = 0.143, 90% Confidence Interval (CI) [0.142, 0.145], and (Standardized Root Mean Square Residual) SRMR = 0.040; standardized loadings ranged from 0.803 to 0.960. In the present item-level dataset, the total scale showed excellent internal consistency (Cronbach’s α = 0.961). The four subscales also showed good to excellent reliability: self-emotion appraisal (α = 0.933), others’ emotion appraisal (α = 0.904), emotion regulation (α = 0.913), and emotion use (α = 0.888). The total parental emotional intelligence score was standardized before use in the main analyses. The elevated RMSEA was interpreted cautiously. In very large samples, small localized discrepancies may become statistically prominent, and RMSEA may perform less favorably in models with relatively limited degrees of freedom. Accordingly, the WLEIS measurement evidence was evaluated jointly across CFI, TLI, RMSEA, SRMR, and standardized loadings rather than based on a single fit index.

#### 2.2.2. Family Climate Questionnaire

Family climate was assessed using a six-item parent-report questionnaire adapted from established measures of family functioning and family environment, including the Family Adaptability and Cohesion Evaluation Scales and the Family Environment Scale (Olson, 1989; Fei et al., 1991; Phillips et al., 1998). The items assessed the overall emotional and relational atmosphere of the family, including open communication among family members, shared responsibility for family affairs, preference for joint versus solitary activities, shared family rules, freedom of expression, and constructive conflict resolution. Five items were positively worded, and one item reflecting preference for solitary activities within the family was reverse-coded. Items were rated on a 4-point scale ranging from 1 (never) to 4 (always), with higher scores indicating a more positive family climate. The one-factor family climate model fit the data adequately, chi-square (9) = 707.385, *p* < 0.001, CFI = 0.990, TLI = 0.984, RMSEA = 0.076, 90% CI [0.071, 0.081], SRMR = 0.024; standardized loadings ranged from 0.179 to 0.807. In the present item-level dataset, the six-item scale showed acceptable internal consistency (Cronbach’s α = 0.720). The total family climate score was standardized before use in the correlation and supplementary contextual analyses.

#### 2.2.3. Parenting Practices

Parenting practices were measured using a 47-item parent-report Chinese parenting questionnaire developed for the current preschool children family survey (Wang, 2020). The scale included six substantive parenting dimensions and one validity-check dimension. The six substantive dimensions were warmth, autonomy support, structure, rejection, chaos, and coercion. Warmth consisted of eight items assessing affectionate and involved parenting behaviors, such as playing with the child and talking with the child about topics of interest. Autonomy support consisted of 10 items assessing reasoning, encouragement of children’s ideas, and respect for children’s initiative. Structure consisted of seven items assessing clear rules, predictable expectations, and consistent guidance. Rejection consisted of six items reflecting emotional unavailability or rejecting responses. Chaos consisted of six items assessing inconsistent, poorly explained, or emotionally disorganized parenting. Coercion consisted of five items assessing harsh or forceful control. Five additional validity-check items were used for data screening and were not included in substantive scale scores.

Items were rated on a 5-point Likert scale ranging from 1 (strongly inconsistent with me) to 5 (strongly consistent with me). Higher scores on warmth, autonomy support, and structure indicated more positive parenting. Higher scores on rejection, chaos, and coercion indicated more negative parenting. The six-factor parenting model showed acceptable fit: chi-square (804) = 60,636.548, *p* < 0.001, CFI = 0.917, TLI = 0.912, RMSEA = 0.075, 90% CI [0.074, 0.075], and SRMR = 0.058; standardized loadings ranged from 0.378 to 0.876. For reliability analysis of the overall parenting scale, negative parenting items were reverse scored so that higher total scores reflected more adaptive parenting. The substantive 42-item parenting scale showed excellent internal consistency (Cronbach’s α = 0.921). Reliability was also high for positive parenting (25 items; α = 0.939) and negative parenting (17 items; α = 0.885). Reliability coefficients for the six parenting dimensions were as follows: warmth (α = 0.839), autonomy support (α = 0.901), structure (α = 0.765), rejection (α = 0.764), chaos (α = 0.749), and coercion (α = 0.661). The six standardized parenting dimensions were used as indicators in the latent profile analysis.

#### 2.2.4. Preschool Children’s Social Competence

Children’s social competence was assessed using a parent-report preschool sociality questionnaire designed for children aged 3 to 6 years (Zhuang, 2019). The questionnaire included 40 items covering four substantive dimensions and one validity-check dimension. According to the questionnaire scoring manual, the substantive dimensions were positive social behavior, emotion regulation, self-awareness, and group norms. Positive social behavior consisted of 11 items assessing behaviors such as cooperation, helping, sharing, and positive peer interaction. Emotion regulation consisted of 6 items assessing children’s ability to manage emotional responses in daily situations. Self-awareness consisted of 10 items assessing children’s understanding of themselves and their own behavior in social contexts. Group norms consisted of 5 items assessing children’s ability to follow collective rules and expectations. Eight validity-check items were used for data screening and were not included in the total social competence score.

Items were rated using a 3-point response format. According to the scoring manual, responses were coded from 1 to 3, with higher scores indicating more advanced social competence. The total social competence score was computed from 32 substantive items. The four-factor child social competence model also showed acceptable fit: chi-square (458) = 11,319.259, *p* < 0.001, CFI = 0.914, TLI = 0.907, RMSEA = 0.042, 90% CI [0.041, 0.043], and SRMR = 0.046; standardized loadings ranged from 0.332 to 0.694. In the present item-level dataset, the total social competence scale showed good internal consistency (Cronbach’s α = 0.849). Reliability for positive social behavior was acceptable (α = 0.776), whereas reliability for self-awareness was modest (α = 0.664). Reliability was relatively low for emotion regulation (α = 0.482) and group norms (α = 0.512). Due to low reliability for the emotion regulation and group norms subscales, we focused our analyses on the global total score of child social competence. All subscale-level results are descriptive only and require careful interpretation.

#### 2.2.5. Socioeconomic Status

Family socioeconomic status (SES) was calculated from paternal and maternal occupation and educational attainment following Shi and Shen (2007). Occupational responses were expanded to nine categories and then recoded into four ordered levels: unemployed or underemployed = 1; service and manual workers = 2; clerical employees and small-scale self-employed workers = 3; and enterprise owners, middle managers, military or police personnel, professionals, and government officials = 4. Parental education was coded on a six-point scale ranging from primary school or below (1) to a master’s degree or above (6). Paternal occupation, maternal occupation, paternal education, and maternal education were separately standardized, summed, and standardized again. Higher composite scores indicated higher family SES.

### 2.3. Data Analysis

Statistical analyses were carried out using IBM SPSS (version 22.0), R (Version 4.5.2) and Mplus 8.3. Data preparation and statistical analyses were conducted using the screened dataset containing 13,398 valid cases.

We first computed descriptive statistics and Cronbach’s α, validated measurement structures via Confirmatory Factor Analysis (CFA), and tested common method bias using Harman’s single-factor test and a common latent method factor model. Pearson correlations and cross-sectional nominal indirect association models with 2000 bootstrap resamples were estimated to examine the associations among parental emotional intelligence, parenting profiles, and child social competence. No causal mediation conclusions were drawn given the cross-sectional design. LPA was conducted on six standardized parenting dimensions, with two-to-five-profile solutions compared on fit criteria, entropy, class size and interpretability. A nominal indirect association model adjusting for socioeconomic status was then constructed to estimate the nominal indirect association through parenting profile membership, combining multinomial and linear regression. Robustness checks included multicollinearity tests, kindergarten-cluster robust standard errors and mother-only subsample analyses. The final sample contained 13,398 valid complete cases. All findings were interpreted cautiously without causal inference due to the single-informant cross-sectional design. Family climate was examined only in supplementary contextual analyses, including bivariate correlations, comparisons across parenting profiles, and an expanded multinomial model in which it was entered as an additional predictor. It was not modeled as an intermediate variable.

## 3. Results

### 3.1. Common Method Bias Test

Because all main variables were collected from caregiver questionnaires, Harman’s single-factor test was conducted as a preliminary assessment of common method bias. All substantive item-level indicators from the parental emotional intelligence scale, family climate questionnaire, parenting questionnaire, and child social competence questionnaire were entered into an unrotated principal component analysis. The analysis included 96 substantive items from 13,398 valid cases. The first unrotated component had an eigenvalue of 19.09 and explained 19.89% of the total variance. The first component did not account for a majority of the total item variance. However, Harman’s single-factor test cannot rule out common reporter bias.

A supplementary common latent method factor model was also estimated. The trait-only model showed mixed fit: *χ*^2^ (4359) = 82,420.394, CFI = 0.862, TLI = 0.855, RMSEA = 0.037, and SRMR = 0.038. Adding an equal-loading common latent method factor produced a very similar model fit: *χ*^2^ (4358) = 82,297.903, CFI = 0.862, TLI = 0.855, RMSEA = 0.037, and SRMR = 0.038. The average standardized loading on the common method factor was 0.116, and the average squared loading was 0.014. These results did not indicate a dominant common method factor. However, because all substantive variables were reported by the same caregiver, these analyses were interpreted as diagnostic checks rather than definitive evidence that common reporter bias was absent.

### 3.2. Descriptive Statistics and Correlations

The final screened sample consisted of 13,398 preschool children and their caregivers. Boys accounted for 52.7% of the sample and girls accounted for 47.3%. The mean age of the children was 4.42 years (SD = 1.13). Most respondents were mothers, and most families lived in urban areas. These descriptive results indicate that the analytic sample was large and geographically broad, although mothers and urban families were overrepresented. Descriptive statistics for the main study variables are reported in Table 1. The raw family climate score ranged from 6 to 24 (M = 18.08, SD = 2.88). Positive parenting was generally high on the 5-point scale (M = 4.14, SD = 0.44), whereas negative parenting was moderate to low (M = 2.57, SD = 0.63). Standardized variables had means close to zero and standard deviations of one, as expected.

Pearson correlations are presented in Table 2. Family climate yielded positive correlations with parental EI, positive parenting, overall parenting quality, and child social competence, while correlating negatively with negative parenting. Parental EI was positively linked to positive parenting and child social competence and negatively linked to negative parenting. Positive parenting correlated positively with child social competence, whereas negative parenting exhibited a negative correlation with child social competence. Socioeconomic status (SES) had a weak negative correlation with child social competence, *r* = −0.041, *p* < 0.001. Given the very large sample, this weak association reached statistical significance despite the negligible magnitude of the association.

The correlation among the contextual and parenting variables was the association between family climate and positive parenting, suggesting that supportive parenting was closely embedded in a warmer family atmosphere. Parental emotional intelligence also showed a moderate positive association with positive parenting, indicating that emotionally competent caregivers were more likely to report supportive parenting behaviors. The small negative correlation between socioeconomic status and child social competence was statistically significant because of the large sample size, but its magnitude was very small; therefore, socioeconomic status was treated as a covariate rather than a central explanatory variable.

### 3.3. Variable-Centered Indirect Associations Through Parenting Practices

A parallel indirect association model was estimated to examine whether positive and negative parenting practices statistically accounted for part of the association between parental EI and child social competence. Parental EI was entered as the predictor, positive and negative parenting were entered simultaneously as intermediate variables, and child social competence was entered as the outcome. Child gender was included as a covariate. As shown in Table 3, parental EI was positively associated with positive parenting and negatively associated with negative parenting. In the outcome model, positive parenting was positively associated with child social competence, whereas negative parenting was negatively associated with child social competence. The direct association between parental EI and child social competence remained significant after parenting variables were included.

**Figure 1:**
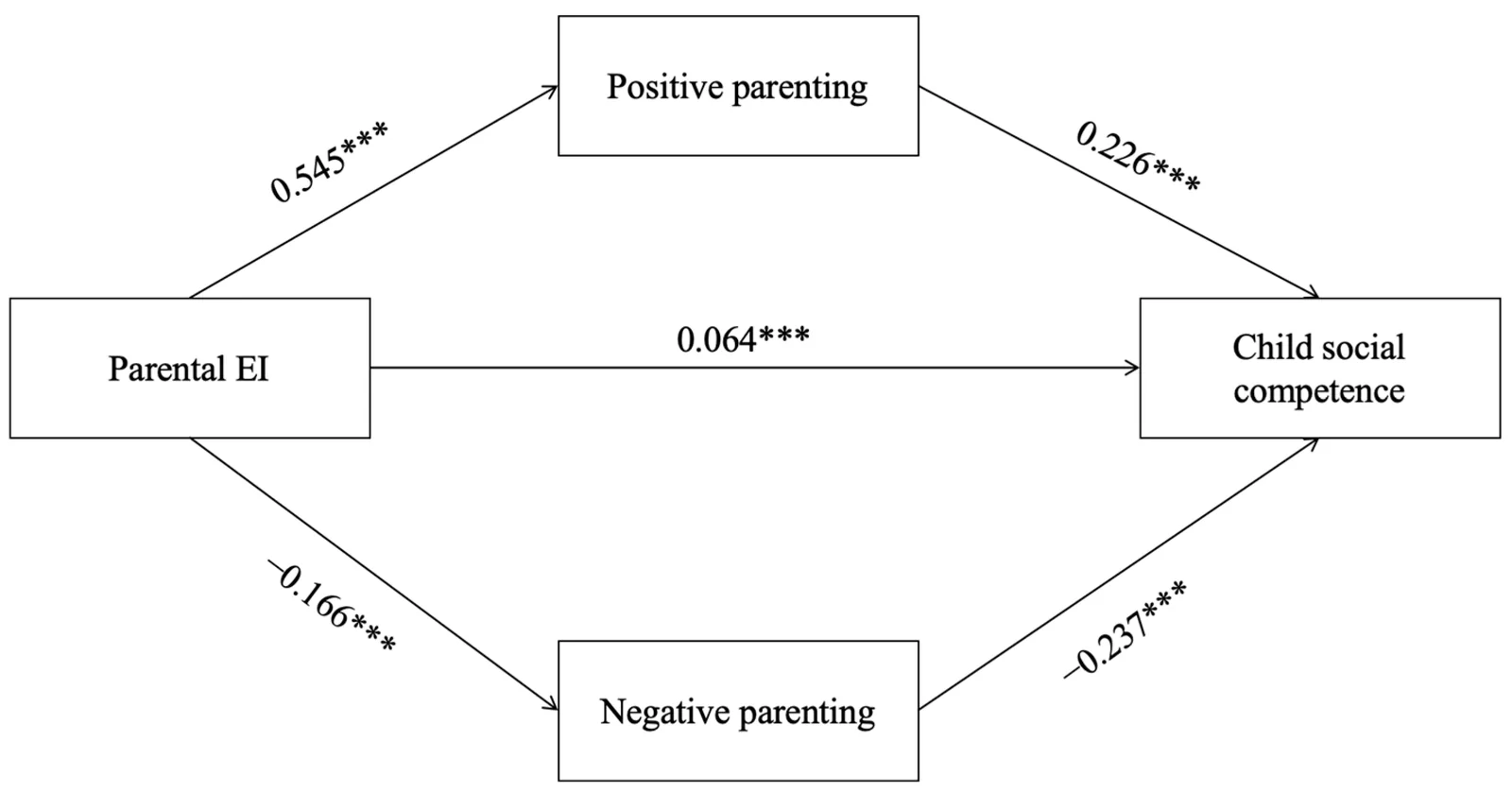
Statistical indirect association model depicting cross-sectional links between parental emotional intelligence, parenting behaviors, and child social competence. Note: *** *p* < 0.001.

Bootstrap analyses further supported the indirect association through both parenting pathways. As shown in Table 4 and Figure 1, the indirect association through positive parenting was 0.123, 95% CI [0.112, 0.135], and the indirect association through negative parenting was 0.039, 95% CI [0.034, 0.045]. The total indirect association was 0.163, 95% CI [0.150, 0.175]. These results indicate that positive and negative parenting statistically accounted for part of the cross-sectional association between parental EI and child social competence.

### 3.4. Gender Differences in the Parenting-Social Competence Pathway

**Figure 2:**
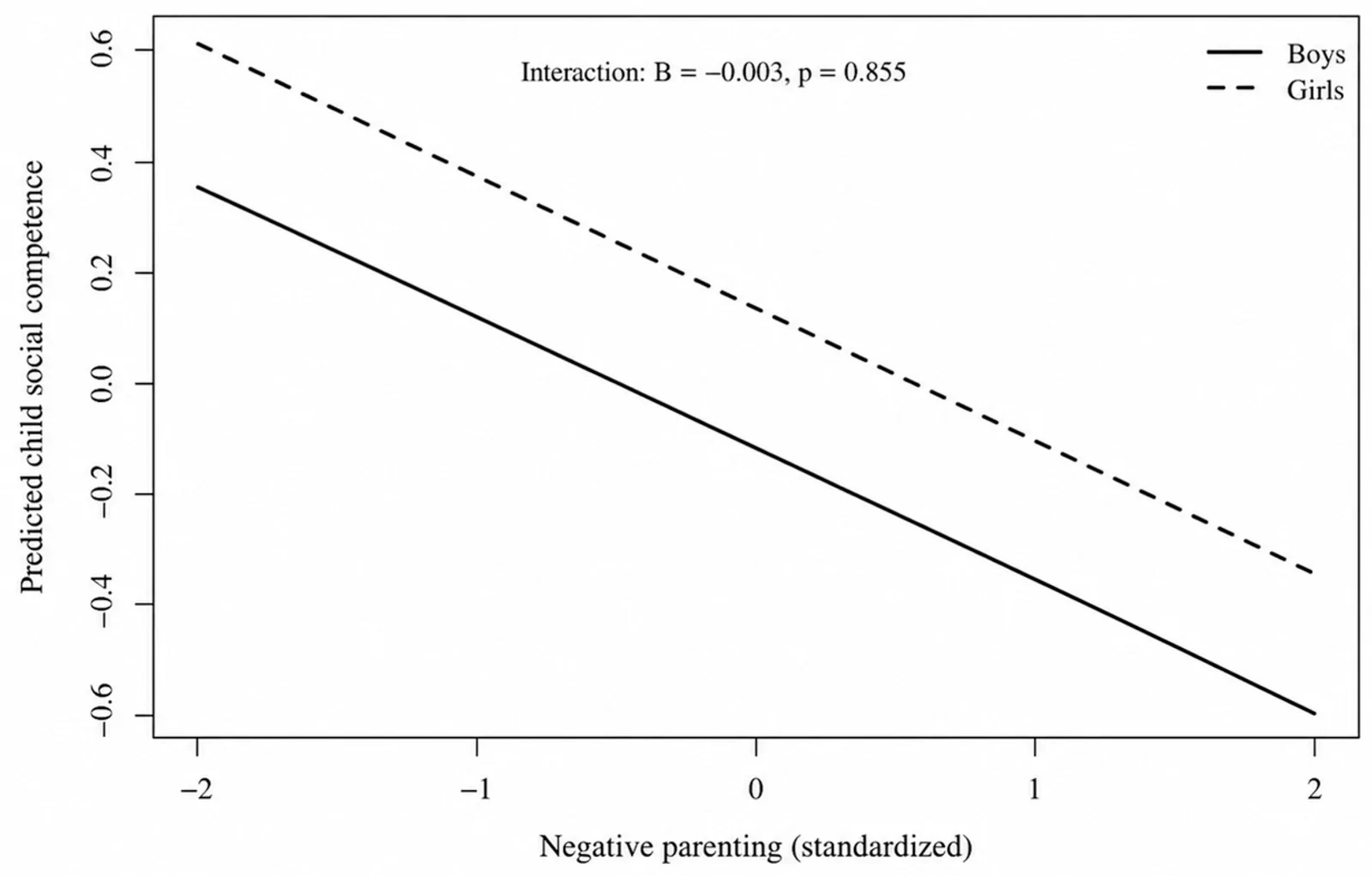
Simple slopes for the association between negative parenting and child social competence by child gender. Note: the interaction was not statistically significant.

Gender moderation was tested by adding child gender interactions with positive parenting and negative parenting to the regression model predicting child social competence. As shown in Table 5, neither the positive parenting by child gender interaction nor the negative parenting by child gender interaction was statistically significant. Thus, the current screened dataset did not support a significant gender-moderated parenting pathway. Positive parenting was positively associated with child social competence for both boys and girls, whereas negative parenting was negatively associated with child social competence for both groups. Figure 2 shows the simple slope pattern for negative parenting by child gender.

The nearly parallel slopes shown in Figure 2 were consistent with the non-significant interaction terms reported in Table 5. Child gender remained included as a theoretically relevant covariate in the main indirect-association analyses; however, the non-significant interactions did not support estimating separate models for boys and girls.

### 3.5. Latent Profile Analysis of Parenting Practices

Latent profile analysis was conducted using the six standardized parenting dimensions: warmth, autonomy support, structure, rejection, chaos, and coercion. Models with two to five profiles were re-estimated in Mplus 8.3 using robust maximum likelihood estimation. The same class-invariant diagonal covariance specification was used across all models: indicator means were freely estimated across profiles, residual variances were constrained to equality across profiles, and within-profile residual covariances were fixed to zero. Each model used 1000 random sets of starting values, with the best 250 solutions retained for final-stage optimization; the initial-stage optimization used 20 iterations. For all reported solutions, the best log-likelihood was replicated 250 times, reducing concern that the retained results reflected an unrecovered local maximum. As shown in Table 6, Akaike information criterion (AIC), Bayesian information criterion (BIC), and sample-size-adjusted BIC decreased as additional profiles were extracted, and the Lo-Mendell-Rubin Adjusted Likelihood Ratio Test (LMR-LRT) and Bootstrap Likelihood Ratio Test (BLRT) were statistically significant across adjacent model comparisons. Classification quality was also acceptable. For the retained four-profile solution, entropy was 0.898, classification error was 0.058, and the class-specific average posterior probabilities were 0.947, 0.914, 0.945, and 0.940. Because the downstream analyses used most-likely class assignments, uncertainty in latent profile classification was not fully propagated into the multinomial and outcome models. The nominal indirect-association analysis should therefore be interpreted as exploratory.

The smallest four-profile class contained 1094 participants (8.16% of the sample). Although the five-profile solution showed lower information criteria and slightly higher entropy (0.907), it produced a very small additional class of 248 participants (1.85%) characterized by simultaneously high positive indicators and extremely high negative indicators, thereby subdividing the mixed high-negative configuration. Considering fit indices, likelihood-ratio tests, classification quality, class size, replicated log-likelihoods, interpretability, and parsimony jointly, the four-profile solution was retained.

**Figure 3:**
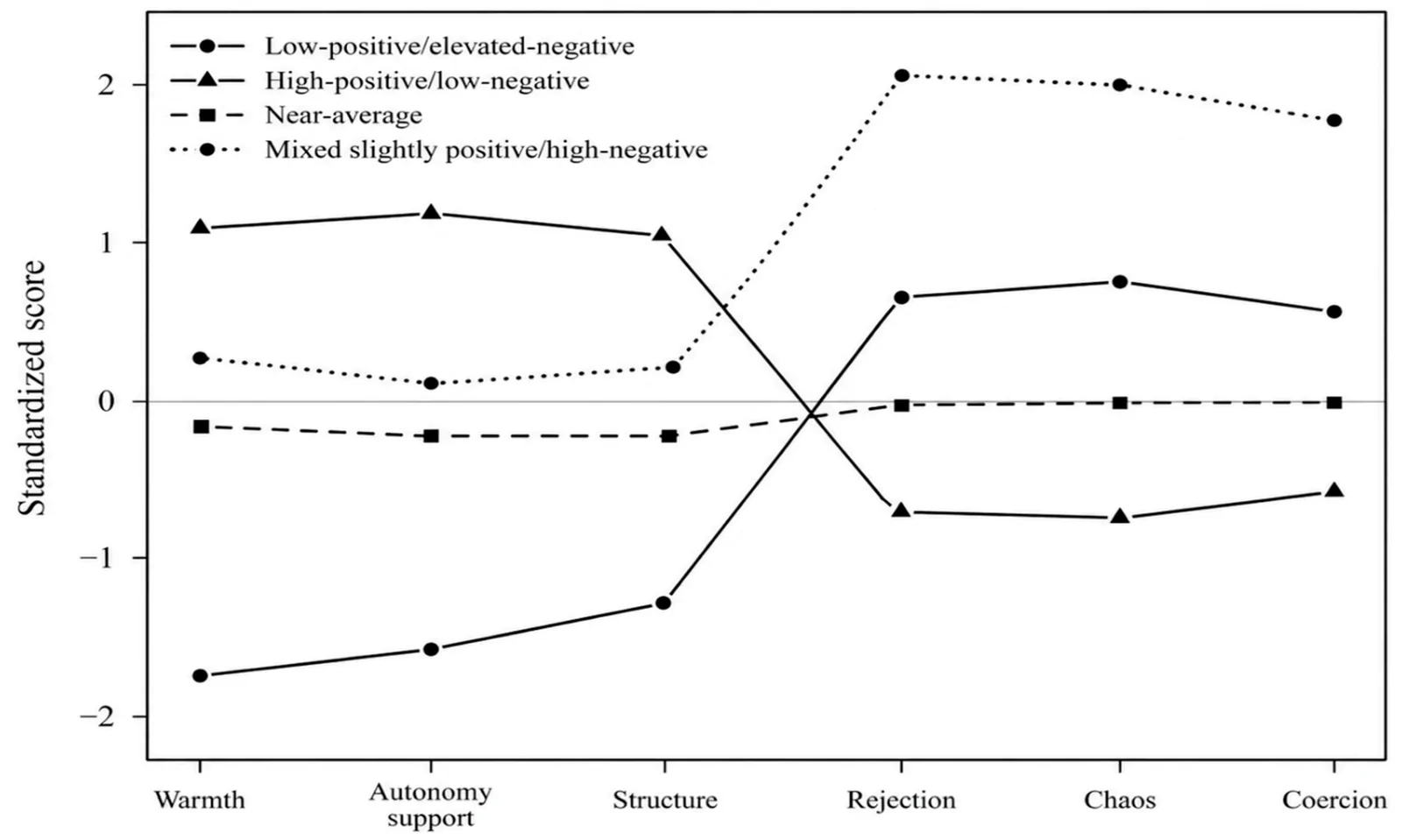
Latent parenting profiles across six standardized parenting indicators.

The four retained profiles are presented in Table 7 and Figure 3. The labels were assigned as descriptive shorthand based on the relative elevation and shape of the six indicators and should not be interpreted as fixed or universally generalizable parenting types. The low-positive/elevated-negative profile included 1227 caregivers (9.16%) and showed very low warmth, autonomy support, and structure, together with elevated rejection, chaos, and coercion. The high-positive/low-negative profile included 3683 caregivers (27.49%) and showed high warmth, autonomy support, and structure and low rejection, chaos, and coercion. The near-average profile included 7394 caregivers (55.19%) and showed slightly below-average positive parenting indicators and near-average negative parenting indicators. The mixed slightly positive/high-negative profile included 1094 caregivers (8.16%) and showed slightly above-average positive parenting indicators but markedly elevated rejection, chaos, and coercion.

The profiles showed both level and shape differences. The high-positive/low-negative and low-positive/elevated-negative profiles largely reflected opposite overall levels of adaptive and maladaptive parenting. In contrast, the mixed slightly positive/high-negative profile showed a less uniform configuration, combining slightly above-average positive indicators with markedly elevated rejection, chaos, and coercion. This mixed pattern provided the clearest evidence that the six parenting dimensions were not fully reducible to a single positive-to-negative continuum.

### 3.6. Nominal Indirect Associations Through Parenting Profiles

Parenting profile membership, based on most-likely class assignment from the retained four-profile solution, was examined as a nominal intermediate variable between parental emotional intelligence and child social competence. Socioeconomic status was included as a covariate, and the low-positive/elevated-negative profile was used as the reference category. As shown in Table 8, higher parental EI was associated with substantially greater odds of membership in the high-positive/low-negative profile rather than the low-positive/elevated-negative profile, Odds Ratio (OR) = 7.355, *p* < 0.001. Higher parental EI was also associated with greater odds of membership in the near-average profile, OR = 1.938, *p* < 0.001, and the mixed slightly positive/high-negative profile, OR = 2.821, *p* < 0.001. Socioeconomic status was positively associated with membership in the high-positive/low-negative and near-average profiles relative to the reference profile, whereas its association with the mixed slightly positive/high-negative profile did not reach statistical significance, *p* = 0.055.

The outcome regression results are presented in Table 9. Relative to children in the low-positive/elevated-negative profile, children in the high-positive/low-negative profile showed the largest difference in social competence, *B* = 1.070, *SE* = 0.034, *p* < 0.001. Smaller but statistically significant differences were observed for the near-average profile, *B* = 0.663, *SE* = 0.030, *p* < 0.001, and the mixed slightly positive/high-negative profile, *B* = 0.334, *SE* = 0.040, *p* < 0.001. Parental EI remained positively associated with child social competence after profile membership and socioeconomic status were included, *B* = 0.119, *SE* = 0.009, *p* < 0.001. Socioeconomic status showed a small negative conditional association with child social competence, *B* = −0.107, *SE* = 0.008, *p* < 0.001. The model accounted for 13.5% of the variance in child social competence.

**Figure 4:**
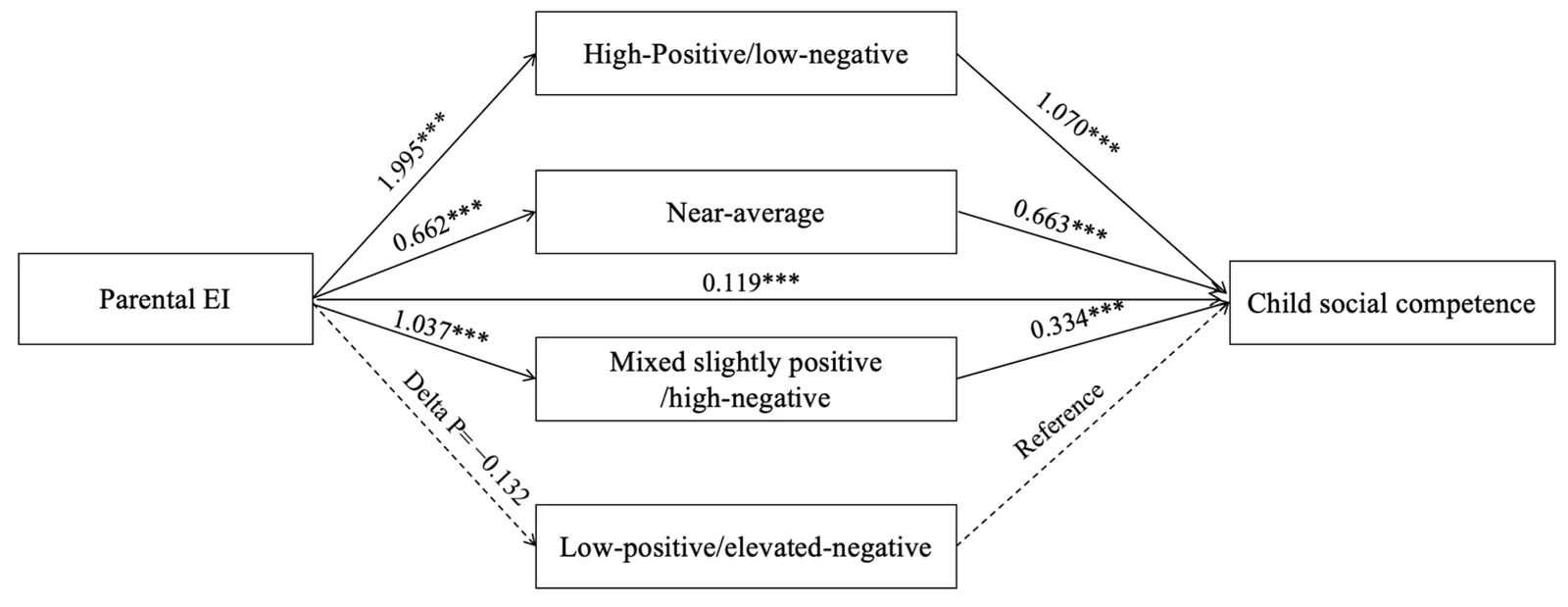
Cross-sectional nominal indirect-association model linking parental emotional intelligence, parenting profiles, and child social competence. Note: Paths from Parental EI to profiles are multinomial logit coefficients relative to the low-positive/elevated-negative reference profile. Profile-to-outcome paths are regression coefficients relative to the reference profile. Socioeconomic status was controlled; Delta P indicates the predicted probability change from low EI (−1 SD) to high EI (+1 SD); Total nominal indirect association = 0.263, 95% bootstrap CI [0.240, 0.286]. *** *p* < 0.001.

The nominal indirect-association decomposition is presented in Table 10 and Figure 4. At parental EI values of −1 SD and +1 SD, the predicted probability of membership in the high-positive/low-negative profile was 0.064 and 0.505, respectively. This probability difference made the largest positive contribution to the total nominal indirect association, contribution = 0.472, 95% CI [0.433, 0.510]. In contrast, the predicted probability of near-average profile membership was lower at +1 SD of parental EI than at −1 SD, producing a negative contribution, contribution = −0.213, 95% CI [−0.236, −0.189]. This negative contribution reflects redistribution of predicted profile probabilities and does not indicate that the near-average profile was less favorable than the reference profile. The contribution of the mixed slightly positive/high-negative profile was close to zero, contribution = 0.004, 95% CI [−0.001, 0.009]. The total nominal indirect association was 0.263, 95% bootstrap CI [0.240, 0.286]. This estimate represents the change in expected standardized child social competence attributable to profile-probability redistribution when parental EI was contrasted at −1 SD versus +1 SD.

### 3.7. Supplementary Contextual Role of Family Climate

Family climate was not treated as the primary predictor in the statistical indirect-association model because the central theoretical question concerned parental EI. However, family climate was retained as an important contextual variable. As shown in Table 2, family climate was positively associated with parental EI, positive parenting, parenting quality, and child social competence, and negatively associated with negative parenting. In addition, profile differences in family climate were large, *F* (3, 13,394) = 1817.703, *p* < 0.001, η^2^ = 0.289. These findings suggest that family climate was closely related to the emotional and parenting processes examined in the main model.

When family climate was included as an additional contextual predictor in an expanded multinomial model, parental EI remained a significant predictor of membership in the high-positive/low-negative profile relative to the low-positive/elevated-negative profile, *OR* = 4.434, *p* < 0.001, although the coefficient was reduced compared with the primary model. This pattern indicates that family climate may share explanatory variance with parental EI and parenting profiles. Therefore, family climate is best interpreted as an important contextual correlate and potential background element rather than as the main independent variable in the present EI-centered model. These supplementary analyses were limited to correlations, profile comparisons, and the expanded multinomial model. We did not estimate a separate indirect-association model with family climate as an intermediate variable because family climate overlaps conceptually with parental EI and parenting practices, and the cross-sectional design could not establish its temporal position.

Sensitivity analyses restricted to mothers (*n* = 10,419) yielded substantively similar patterns. The indirect associations through positive and negative parenting were 0.118, 95% CI [0.104, 0.132], and 0.047, 95% CI [0.040, 0.054], respectively, and the remaining direct association was 0.066, 95% CI [0.043, 0.088]. The profile proportions and the directions of the profile-membership and outcome-model coefficients were also comparable with those observed in the full sample.

## 4. Discussion

The present study examined whether parental emotional intelligence was associated with preschool children’s social competence through two complementary parenting routes: variable-centered parenting practices and person-centered parenting profiles. The results converged on a coherent pattern. Parents with higher EI reported more positive parenting, less negative parenting, and a greater likelihood of belonging to the high-positive/low-negative profile; in turn, these parenting patterns were associated with higher child social competence. This does not establish a developmental causal pathway, because all variables were measured at one time point and reported by caregivers. Nevertheless, the findings indicate that parental EI may be meaningful not only as an individual emotional resource but also as a family-level behavioral pattern expressed through everyday caregiving.

This interpretation is consistent with emotion socialization theory, which holds that children’s socioemotional development is shaped by repeated exposure to parents’ emotional expressiveness, responses to children’s emotions, and emotion-related communication (Eisenberg et al., 1998; Morris et al., 2007). Ability-based and self-report research has shown that emotional abilities are associated with social functioning, relationship quality, and adaptive interaction patterns (MacCann & Roberts, 2008). It also extends recent work showing that parental emotional abilities are linked to children’s adjustment, mental health, and emotion-related outcomes (Cabello et al., 2023; Romero González et al., 2021). Emotionally competent caregivers may be better able to recognize children’s emotional cues, regulate their own responses during conflict, and provide guidance that combines emotional support with behavioral structure. The present findings therefore add to prior literature by showing that these associations can be observed both at the level of discrete parenting dimensions and at the level of naturally co-occurring parenting configurations.

### 4.1. Parental EI and the Behavioral Parenting Pathway

The variable-centered results clarify one behavioral route through which parental EI was associated with children’s social competence. Parental EI was positively associated with positive parenting and negatively associated with negative parenting, and both parenting dimensions were uniquely associated with child social competence. This pattern is consistent with Belsky’s process model of parenting, which proposes that parental psychological resources shape parenting behavior and, through parenting, children’s development (Belsky, 1984). It also fits recent developmental work showing that warmth, autonomy support, and structure can support children’s emotion regulation, effortful control, and prosocial behavior, whereas rejection, coercion, and chaotic discipline may undermine regulatory and interpersonal capacities (Morris et al., 2017; Taylor et al., 2013).

The larger indirect association through positive parenting is theoretically informative. It suggests that social competence in early childhood may depend not only on the absence of harsh or inconsistent parenting but also on the active presence of supportive, autonomy-enhancing, and structured practices. This interpretation is consistent with intervention evidence showing that emotion-focused parenting programs can improve parental emotion socialization and young children’s emotional and behavioral adjustment (England-Mason et al., 2023; Havighurst et al., 2022). From this perspective, parental EI may be linked to child social competence because it helps caregivers translate emotional awareness and regulation into concrete behaviors, such as listening to children’s feelings, explaining rules calmly, providing predictable routines, and reducing coercive escalation.

### 4.2. Parenting Profiles and the Contribution of a Person-Centered Approach

The person-centered findings extend the variable-centered results by showing how parenting practices were organized within families. Four descriptive profiles were retained: high-positive/low-negative, near-average, low-positive/elevated-negative, and mixed slightly positive/high-negative. The first and third profiles largely reflected opposite overall levels of adaptive and maladaptive parenting. Children in the high-positive/low-negative profile showed the highest social competence, whereas children in the low-positive/elevated-negative profile showed the lowest. These results are consistent with person-centered studies suggesting that parenting and emotion socialization profiles can capture family processes that may be obscured when parenting variables are examined separately (Arikan & Kumru, 2023; Howe & Zimmer-Gembeck, 2022; King et al., 2023; Trevethan et al., 2021).

At the same time, the profile solution should not be overread as a fixed taxonomy of Chinese parenting. The near-average profile was best understood as a large, sample-relative group located close to the overall mean rather than as a normatively ideal or qualitatively distinct parenting type. Likewise, the mixed slightly positive/high-negative profile was not simply a midpoint between positive and negative parenting. It showed a different shape, combining slightly above-average positive indicators with markedly elevated rejection, chaos, and coercion. This mixed pattern provided the clearest evidence that parenting practices were not fully reducible to a single positive-to-negative continuum. The contribution of LPA, therefore, lies less in replacing variable-centered analysis and more in showing how the same parenting dimensions may cluster into family-level configurations with different developmental correlates.

### 4.3. Interpreting the Mixed Slightly Positive/High-Negative Profile

The mixed slightly positive/high-negative profile warrants cautious interpretation because it combines seemingly contradictory features. Caregivers in this group reported slightly above-average warmth, autonomy support, and structure, but also very high rejection, chaos, and coercion. This pattern should not be described as supportive or adaptive. Rather, it may reflect a form of high involvement in which some organization and engagement coexist with emotionally harsh, inconsistent, or coercive practices. In Chinese family contexts, high parental involvement and firm control may sometimes be framed as responsibility, investment, or concern; however, when these behaviors are accompanied by rejection, coercion, and chaotic discipline, their developmental meaning may become less protective (Chao, 2000; Friedlmeier et al., 2011).

Chinese cultural values emphasizing family harmony, relational interdependence, respect for parental authority, and expectations of children’s obedience may shape how structure and control are understood and reported. In some families, firm guidance may be interpreted as responsible involvement intended to preserve harmony and socialize appropriate conduct. However, these cultural meanings do not imply that rejection, coercion, or chaotic discipline are benign. The identified configurations may not generalize directly to cultural settings in which autonomy, obedience, parental authority, and family harmony carry different meanings; the prevalence and interpretation of the mixed slightly positive/high-negative profile, in particular, may differ across societies.

Children in the mixed slightly positive/high-negative profile showed higher social competence than those in the low-positive/elevated-negative profile but lower competence than those in the high-positive/low-negative profile. This pattern allows a balanced interpretation. On the one hand, the presence of some warmth, structure, or autonomy support may be associated with better social functioning than a broadly low-positive/elevated-negative configuration. On the other hand, slightly positive parenting indicators did not appear to offset the risk associated with very high rejection, chaos, and coercion. The profile therefore highlights a clinically and educationally relevant point: parent education should not only encourage warmth and involvement, but also address coercive discipline, inconsistent rules, and emotionally rejecting responses.

### 4.4. Nominal Indirect Associations and Probability Redistribution

The nominal indirect-association model further clarified how parental EI was linked to profile membership. Higher parental EI substantially increased the probability of belonging to the high-positive/low-negative profile, which was also the profile most strongly associated with child social competence. At the same time, higher parental EI reduced the probability of membership in the low-positive/elevated-negative and near-average profiles. This probability shift suggests that parental EI may be associated not only with more positive parenting on average but also with a greater likelihood that parenting practices are organized into a broadly adaptive configuration.

The negative contribution of the near-average profile in the decomposition should be interpreted statistically rather than substantively. It does not mean that near-average parenting was harmful relative to the low-positive/elevated-negative profile. Rather, the negative value reflects probability redistribution: as parental EI increased, families with higher parental EI had a lower predicted probability of classification in the near-average profile and a higher predicted probability of classification in the high-positive/low-negative profile. This distinction is important because latent profile labels can easily be reified. Consistent with recommendations for latent class and latent profile models, the profiles should be treated as descriptive summaries of observed response patterns rather than stable family types (Nylund et al., 2007).

### 4.5. SES and Family Climate as Contextual Findings

The SES findings were more complex than expected. In the multinomial models, higher SES was associated with greater odds of membership in the high-positive/low-negative profile relative to the low-positive/elevated-negative profile, which is consistent with family stress and investment perspectives suggesting that socioeconomic resources may support more adaptive parenting environments (Bradley & Corwyn, 2002; Conger & Donnellan, 2007). However, after parental EI, parenting profiles, and covariates were included, SES showed a small negative association with child social competence. This result is counterintuitive and should be interpreted with restraint. Diagnostics suggested that the association was unlikely to be explained solely by multicollinearity, but alternative explanations remain plausible.

One possibility is that the residual component of SES, after family emotional and parenting processes were controlled, captured stricter parental expectations or reporting standards among higher-SES caregivers. Another possibility is that the SES composite did not fully distinguish parental education, occupational demands, material resources, and neighborhood context. Because the effect size was small and the design was cross-sectional, the result should not be interpreted as evidence that socioeconomic advantage harms children’s social competence. Instead, it points to the need for more refined SES measurement in future studies.

Family climate also emerged as an important contextual correlate, but it was not positioned as the primary explanatory pathway in this study. Family climate was positively associated with parental EI, parenting quality, and child social competence, and it differed across parenting profiles. These results are compatible with emotional security and family emotion socialization perspectives, which emphasize that children’s social development unfolds within the broader affective atmosphere of family life (Cummings & Davies, 1994; Morris et al., 2007). Cross-cultural research also indicates that emotion socialization is embedded in cultural values and family interaction patterns, suggesting that studies in Chinese preschool families can contribute to a more contextually sensitive understanding of how emotional family processes support social development (Labella, 2018). When family climate was included in supplementary models, the association between parental EI and high-positive/low-negative profile membership was reduced but remained significant. This pattern suggests shared variance among parental EI, family climate, and parenting practices. Future longitudinal research should examine whether family climate functions as an antecedent, mediator, moderator, or parallel contextual process.

### 4.6. Limitations and Future Directions

Several limitations should be acknowledged. First, the cross-sectional design prevents causal inference. Although the model was theoretically organized from parental EI to parenting and child social competence, reciprocal processes are plausible: children’s social competence may shape parenting behavior, and caregivers’ perceptions of their own EI may link to how they evaluate both parenting and child functioning. Cross-sectional indirect associations can be biased when interpreted as longitudinal mediation, so the present findings should be described as statistical indirect associations rather than causal mechanisms (Maxwell & Cole, 2007).

An informative next step would be a three-wave design with approximately six-month intervals. Parental EI, parenting practices, profile indicators, and child social competence could be assessed at T1; parenting practices and profile indicators could be reassessed at T2; and child social competence could be reassessed at T3. Longitudinal indirect-association models controlling for baseline parenting and child competence, together with latent transition analysis of repeated profile indicators, would provide a stronger test of temporal ordering and profile stability.

In addition, all main variables were reported by caregivers, which may introduce shared reporter variance. The common method checks did not indicate a dominant method factor, but such analyses cannot eliminate common reporter bias. Methodological reviews have emphasized that common method bias is multifaceted and difficult to eliminate through post hoc statistical procedures alone (Podsakoff et al., 2003, 2024). Future studies should incorporate teacher ratings, observational measures of parent–child interaction, behavioral tasks of child social competence, and longitudinal designs. Although scale-level CFAs were added in the present revision, some measurement evidence was mixed: the WLEIS model showed an elevated RMSEA despite strong comparative fit and high loadings, the brief family climate model included one low-loading item, and the parenting questionnaire requires further validation of its factor structure and measurement equivalence across respondent types. Several child social competence subscales showed low reliability, especially emotion regulation and group norms. For this reason, the total social competence score should be treated as the primary outcome, whereas subscale-level interpretations require caution. Although the mother-only analyses supported the robustness of the main pattern, they did not establish measurement invariance across caregiver types. Future research should formally test configural, metric, and scalar invariance across mothers and fathers because systematic differences in parental perceptions may affect both scale scores and profile classification.

The identified profiles should be interpreted as exploratory and sample-specific configurations. Although the large sample increased estimation precision, latent profiles may vary across samples, instruments, model specifications, and respondent composition. The present sample also predominantly consisted of mothers and urban families. The mother-only robustness check produced broadly similar results, but it did not establish measurement invariance across mothers and fathers. Replication with father reports, observational data, more socioeconomically diverse families, and independent samples is needed before the four-profile structure can be treated as reproducible. The four profiles should not be interpreted as stable parenting typologies; they remain provisional until their number, shape, prevalence, and correlates are replicated across independent samples, cultural settings, alternative measurement instruments, and repeated longitudinal assessments. Future studies should combine multi-informant reports and observational assessments with formal measurement-invariance tests across respondent groups and cultural settings. Repeated profile assessments and latent transition analysis are also needed to determine whether these configurations remain stable or change over time.

### 4.7. Contributions and Practical Implications

Despite these limitations, the study makes several contributions. Theoretically, it integrates emotional intelligence theory, emotion socialization theory, and person-centered parenting analysis to explain preschool children’s social competence. Empirically, it shows that parental EI is associated with child social competence through two complementary routes: a variable-centered route involving positive and negative parenting practices and a person-centered route involving parenting profiles. Methodologically, the use of LPA highlights that parenting practices may form heterogeneous configurations, including a mixed slightly positive/high-negative profile that would be difficult to detect using only mean-level associations.

The practical implications should remain modest. The findings suggest that parent-focused interventions may benefit from helping caregivers translate emotional competence into concrete parenting behaviors, especially warmth, autonomy support, structure, and reduced coercive or chaotic discipline. In Chinese kindergarten-based parent education, such content could be incorporated into parent meetings, family education workshops, and home-kindergarten collaboration activities through brief modules on emotion coaching, reflective listening, non-coercive limit setting, and consistent routines. These suggestions should not be presented as intervention evidence from the present study. Rather, they identify plausible directions for future programs that can be tested in longitudinal or experimental designs.

Potential intervention targets within parental EI include recognizing one’s own emotional states, accurately perceiving children’s emotional cues, regulating frustration during difficult interactions, and using emotional information constructively in problem solving. Families in the low-positive/elevated-negative profile may require combined work on warmth, autonomy support, predictable structure, and reductions in rejection and coercion. For families in the near-average profile, low-intensity preventive support may focus on strengthening consistent positive practices. Families in the mixed slightly positive/high-negative profile may benefit particularly from reducing harshness, inconsistency, and emotional rejection while preserving constructive involvement and structure. Families in the high-positive/low-negative profile may benefit primarily from maintenance and reinforcement strategies. These profile-informed recommendations are provisional, should not be used as diagnostic labels, and require direct evaluation in longitudinal or experimental intervention studies.

In summary, parental EI was associated with preschool children’s social competence through both parenting practices and parenting profiles. Higher EI was linked to more positive and less negative parenting, and it was also associated with a greater probability of belonging to the high-positive/low-negative profile. The mixed slightly positive/high-negative profile further shows that slightly positive and highly negative parenting indicators may coexist within some families, underscoring the value of a cautious person-centered approach. Because the evidence is cross-sectional and caregiver-reported, the findings should be understood as theoretically informed associations rather than causal pathways.

## 5. Conclusions

The present study suggests that parental emotional intelligence is associated with preschool children’s social competence through both parenting practices and parenting profiles. Higher parental EI was linked to more positive and less negative parenting, and these practices statistically accounted for part of the association between parental EI and child social competence. In addition, higher parental EI was associated with a greater probability of belonging to the high-positive/low-negative profile, which showed the highest level of child social competence. The mixed slightly positive/high-negative profile further indicates that slightly elevated positive parenting practices and highly negative parenting practices may coexist within some families.

Overall, the findings support the view that parental EI may be developmentally meaningful when it is expressed through organized patterns of caregiving. However, because the data were cross-sectional and caregiver-reported, the results should be interpreted as evidence of theoretically informed associations rather than causal pathways. Future studies should combine three-wave longitudinal designs with reports from mothers, fathers, and teachers and with observational assessments. They should test measurement invariance across respondent groups and cultural settings, examine longitudinal indirect associations while controlling prior levels, and use latent transition analysis to evaluate whether parenting profiles remain stable or change over time.

## Figures and Tables

**Table 1:** Descriptive statistics (total *n* = 13,398).

Variable	M	SD	Min	Max	Skew	Kurtosis
Family climate, raw total	18.075	2.877	6	24	−0.051	0.064
Positive parenting, raw mean	4.139	0.44	1	5	−0.405	2.247
Negative parenting, raw mean	2.573	0.628	1	5	0.754	1.326
*Z* Family climate	0	1	−4.197	2.06	−0.051	0.061
*Z* Parental emotional intelligence	0	1	−5.25	1.398	−1.392	3.867
*Z* Positive parenting	0	1	−7.14	1.958	−0.405	2.247
*Z* Negative parenting	0	1	−2.506	3.865	0.754	1.326
*Z* Parenting quality	0	1	−3.839	2.771	0.046	−0.282
*Z* Child social competence	0	1	−5.622	2.392	−0.236	−0.133
*Z* Positive social behavior	0	1	−4.776	1.827	−0.319	−0.177
*Z* Emotion regulation	0	1	−4.287	1.686	−0.39	−0.275
*Z* Self-awareness	0	1	−3.83	2.209	−0.166	−0.281
*Z* Group norms	0	1	−4.538	1.671	−0.375	−0.141
*Z* Socioeconomic status	0	1	−3.672	1.252	−0.95	0.175

**Table 2:** Correlations among key variables.

Variable	1	2	3	4	5	6	7
1. Socioeconomic status	--						
2. Family climate	0.142 ***	--					
3. Parental EI	0.163 ***	0.392 ***	--				
4. Positive parenting	0.147 ***	0.589 ***	0.545 ***	--			
5. Negative parenting	−0.090 ***	−0.319 ***	−0.166 ***	−0.298 ***	--		
6. Parenting quality	0.147 ***	0.564 ***	0.442 ***	0.805 ***	−0.805 ***	--	
7. Child social competence	−0.041 ***	0.304 ***	0.227 ***	0.334 ***	−0.319 ***	0.405 ***	--

Note: All variables in this table are standardized variables, *** *p* < 0.001.

**Table 3:** Path coefficients for the cross-sectional parallel indirect-association model.

Model Path	*B*	*SE*	*t*	*p*	Model *R*^2^
Parental EI → positive parenting	0.545	0.007	75.31	<0.001	0.298
Parental EI → negative parenting	−0.166	0.009	−19.48	<0.001	0.029
Positive parenting → child social competence	0.226	0.01	23.485	<0.001	0.183
Negative parenting → child social competence	−0.237	0.008	−29.001	<0.001	0.183
Parental EI → child social competence, direct association	0.064	0.009	6.861	<0.001	0.183
Parental EI → child social competence, total association	0.227	0.008	27.196	<0.001	0.07

Note: All continuous variables were standardized. Child gender was included as a covariate in all models.

**Table 4:** Bootstrap indirect associations of parental emotional intelligence with child social competence through parenting practices.

Indirect Pathway	Estimate	95% CI Lower	95% CI Upper
Parental EI → Positive parenting → Child social competence	0.123	0.112	0.135
Parental EI → Negative parenting → Child social competence	0.039	0.034	0.045
Total indirect association	0.163	0.150	0.175

Note: Confidence intervals were estimated using 2000 bootstrap resamples.

**Table 5:** Gender moderation in the parenting-social competence pathway.

Predictor	*B*	*SE*	*t*	*p*
Parental emotional intelligence	0.064	0.009	6.861	<0.001
Positive parenting	0.232	0.012	19.270	<0.001
Negative parenting	−0.236	0.011	−21.701	<0.001
Child gender	0.252	0.016	16.068	<0.001
Positive parenting × child gender	−0.014	0.017	−0.825	0.409
Negative parenting × child gender	−0.003	0.017	−0.183	0.855

Note: Boys were coded 0 and girls were coded 1. All continuous variables were standardized. Model *R*^2^ = 0.183.

**Table 6:** Latent profile model fit indices.

Profiles	LL	AIC	BIC	SABIC	Entropy	LMR-LRT *p*	BLRT *p*	Class Proportions
2	−104,854.714	209,747.429	209,889.983	209,829.603	0.842	<0.001	<0.001	0.35893/0.64107
3	−99,496.180	199,044.361	199,239.435	199,156.810	0.869	<0.001	<0.001	0.56904/0.34901/0.08195
4	−95,356.359	190,778.719	191,026.313	190,921.442	0.898	<0.001	<0.001	0.27489/0.09158/0.55187/0.08165
5	−92,958.563	185,997.126	186,297.241	186,170.125	0.907	<0.001	<0.001	0.07031/0.27631/0.52762/0.10725/0.01851

Note. AIC = Akaike information criterion; BIC = Bayesian information criterion; SABIC = sample-size-adjusted Bayesian information criterion; LL = Log-Likelihood; Models were estimated in Mplus 8.3 using Robust Maximum Likelihood Estimation (MLR) with STARTS = 1000 250 and STITERATIONS = 20. The best log-likelihood was replicated 250 times for each model. BLRT was estimated with LRTSTARTS = 0 0 500 100.

**Table 7:** Class-specific standardized parenting profiles.

Parenting Profile	*n*	%	Warmth	Autonomy Support	Structure	Rejection	Chaos	Coercion
Low-positive/elevated-negative	1227	9.16	−1.750	−1.653	−1.373	0.664	0.729	0.542
High-positive/low-negative	3683	27.49	1.040	1.132	0.999	−0.714	−0.750	−0.601
Near-average	7394	55.19	−0.258	−0.301	−0.298	−0.056	−0.044	−0.054
Mixed slightly positive/high-negative	1094	8.16	0.229	0.093	0.201	2.002	1.963	1.749

Note: Values are standardized means. Positive values indicate scores above the sample mean, and negative values indicate scores below the sample mean.

**Table 8:** Multinomial logistic regression of parenting profile membership on parental emotional intelligence and SES.

Contrast	Predictor	*B*	*SE*	*z*	*p*	*OR*
High-positive/low-negative vs. Low-positive/elevated-negative	Intercept	1.114	0.044	25.386	<0.001	3.046
High-positive/low-negative vs. Low-positive/elevated-negative	Parental EI	1.995	0.043	45.953	<0.001	7.355
High-positive/low-negative vs. Low-positive/elevated-negative	SES	0.338	0.035	9.606	<0.001	1.402
Near-average vs. low-positive/elevated-negative	Intercept	2.185	0.039	56.038	<0.001	8.890
Near-average vs. low-positive/elevated-negative	Parental EI	0.662	0.029	23.068	<0.001	1.938
Near-average vs. low-positive/elevated-negative	SES	0.240	0.029	8.230	<0.001	1.272
Mixed slightly positive/high-negative vs. Low-positive/elevated-negative	Intercept	0.268	0.048	5.547	<0.001	1.307
Mixed slightly positive/high-negative vs. Low-positive/elevated-negative	Parental EI	1.037	0.048	21.808	<0.001	2.821
Mixed slightly positive/high-negative vs. Low-positive/elevated-negative	SES	0.077	0.040	1.920	0.055	1.080

Note: parental EI and socioeconomic status were standardized. *OR* = odds ratio.

**Table 9:** Linear regression predicting child social competence from parenting profiles, parental emotional intelligence and SES.

Predictor	*B*	*SE*	*t*	*p*
Intercept	−0.688	0.028	−24.408	<0.001
High-positive/low-negative profile	1.070	0.034	31.421	<0.001
Near-average profile	0.663	0.030	22.250	<0.001
Mixed slightly positive/high-negative profile	0.334	0.040	8.361	<0.001
Parental emotional intelligence	0.119	0.009	13.111	<0.001
Socioeconomic status	−0.107	0.008	−13.034	<0.001

Note: the low-positive/elevated-negative profile was the reference group. Child social competence, parental emotional intelligence, and socioeconomic status were standardized. Model *R*^2^ = 0.135.

**Table 10:** Nominal indirect-association decomposition for parental emotional intelligence and child social competence.

Parenting Profile	P at Low Parental EI	P at High Parental EI	Delta P	B to Child Social Competence	Contribution	95% CI for Contribution
Low-positive/elevated-negative	0.155	0.023	−0.132	0.000	0.000	[0.000, 0.000]
High-positive/low-negative	0.064	0.505	0.441	1.070	0.472	[0.433, 0.510]
Near-average	0.710	0.389	−0.321	0.663	−0.213	[−0.236, −0.189]
Mixed slightly positive/high-negative	0.072	0.083	0.012	0.334	0.004	[−0.001, 0.009]

Note: low and high parental EI represent −1 SD and +1 SD, respectively, with socioeconomic status fixed at the sample mean. The total nominal indirect association was 0.263, 95% bootstrap CI [0.240, 0.286].

## Data Availability

The data that support the findings of this study are available from the corresponding author upon reasonable request. The data are not publicly available due to privacy and ethical restrictions.

## Abbreviations

The following abbreviations are used in this manuscript:

AIC	Akaike Information Criterion
APP	Average Posterior Probability
BIC	Bayesian Information Criterion
BLRT	Bootstrap Likelihood Ratio Test
CFA	Confirmatory Factor Analysis
CFI	Comparative Fit Index
CI	Confidence Interval
EI	Emotional Intelligence
LL	Log-Likelihood
LMR-LRT	Lo-Mendell-Rubin Adjusted Likelihood Ratio Test
LPA	Latent Profile Analysis
MLR	Robust Maximum Likelihood Estimation
OR	Odds Ratio
RMSEA	Root Mean Square Error of Approximation
SABIC	Sample-Size-Adjusted Bayesian Information Criterion
SD	Standard Deviation
SE	Standard Error
SES	Socioeconomic Status
SPSS	Statistical Package for the Social Sciences
SRMR	Standardized Root Mean Square Residual
TLI	Tucker–Lewis Index
WLEIS	Wong and Law Emotional Intelligence Scale
